# The effect of schedule management empowerment on hospital attractivity and nurses’ loyalty: a mixed method study

**DOI:** 10.1186/s12912-026-04713-w

**Published:** 2026-05-25

**Authors:** Sophie Pajoux, Valerie Loizeau

**Affiliations:** 1 Université de Poitiers, CHU de Poitiers, CGS, Poitiers, F-86000 France; 2 Chair in Research, Innovation and Care Practices, Assistance Publique- Hôpitaux de Paris Education and Health Promotion Laboratory (LEPS UR3412), Sorbonne Paris Nord University, Bobigny, France

**Keywords:** Schedule management empowerment, Hospital attractivity, Nurses’ loyalty, Mixed method

## Abstract

**Context:**

Thoroughgoingly transformed by successive crises, particularly Covid-19, medical and nursing organizations have found themselves subject to increasing imbalance. Loyalty building and professional attractivity have come to depend on the confidence, autonomy, and responsibility granted to employees. The cruciality of the physician/head nurse tandem in professional quality of life and patient care is now widely recognized. That much said, as demonstrated in 2020 by the World Health Organization (WH0), scheduling management has remained complex and time-consuming, and continues to represent a major managerial challenge, exacerbated by absenteeism, disinvestment and loss of caregiver motivation. Individual expectations, generational diversity and the quest for self-governance render management tasks even more sensitive.

**Methods:**

Conducted in France in 2024, this explanatory sequential mixed methods study of 208 hospital-based nurses is aimed at measuring the effect of collaborative schedule management on nurses’ satisfaction, and investment.

**Results:**

The results demonstrate that nurses’ active participation in schedule preparation leads to increased satisfaction. Empowerment and collective discipline reinforce their satisafaction and involvement, thereby rendering collaborative management a lever for attractiveness and loyalty building.

**Discussion:**

The study lends perspective to the notion of collective governance, which arose during the 1970s. As a source of collective and individual motivation, team empowerment necessitates progressive acculturation. An enabling environment allying adaptability and flexibility is of paramount importance, and the front-line manager assumes a central role. Models such as the “Magnet-designated Hospital” illustrate the effectiveness of collaborative management as a means of optimizing the nurse’s time management. By favouring autonomy and responsibility, collaborative management has become an attractivity factor conducive to loyalty building in the hospital sector.

**Supplementary Information:**

The online version contains supplementary material available at 10.1186/s12912-026-04713-w.

## Introduction

The different hospital reforms in France have engendered structural modifications impacting patient care pathways and the professional and personal lives of nurses. Resulting imbalances were exacerbated following the Covid-19 pandemic [1], which led to heightened turnover, loss of attractivity and lessened meaningfulness of professional activities [2]. The pandemic constitutes a pivotal component of the framework, as it has profoundly reconfigured the parameters of health, location, time, and governance in the workplace [3]. The pandemic had cascading effects and repercussions on management, two examples being fragmented work time and the introduction of technological advances of possible help in the execution of workaday tasks [4].

A need to attract and retain nurses is fundamental to present-day management, especially in view of the alarming projections of the World Health Organization, which in 2020 estimated a shortage of six million nurses apt to address the needs of the population [5].

Attractivity and loyalty-building are closely associated with confidence, autonomy and responsibility in a secure environment [6]. Management style has an effect on the prevailing climate in a clinical unit and, more precisely, on the capacity for action [7]. In a clinical unit in a hospital structure, management revolves around the doctor – head nurse tandem, which ensures satisfactory quality of work life and quality of patient care [8]. The head nurse is situated in the heart of healthcare organization, like a fulcrum, at the centre of a quadrature formed by patients, physicians, care-provider teams and administrators. Though without specialized expertise, her ability to translate expressed or unexpressed needs endows the head nurse with a fundamental role in the organization of treatment procedures and follow-up [9]. She is tasked with conducting a multitude of caregiving projects [10], rendering her a managerial lever charged with creating meaning and steering teams toward essential and institutionally programmed reflection [11]. That much said, managing the schedules of a team of paramedical professionals in a healthcare unit can’t help but take up a major portion of the head nurse’s daily schedule [12], leaving little place for project management. It has been repeatedly noted that caregiver needs have evolved toward requests for increasingly flexible and largely individualized timetables [13]. As a result, tensions have appeared between pressing demands and continuity of care for hospitalized patients. The role of the head nurse in ensuring time management compatible with care loads is fundamental [7], and schedule management is additionally complicated by high absenteeism rates, which are likely to negatively impact team motivation [1, 10]. Caregiver disinvestment and a loss of workplace meaning are realities underscoring an increased need for loyalty building and professional investment [14].

Workplace organisation and work time are defined in various countries by the applicable laws [15]. In each clinical unit of a given hospital, the head nurse determines daytime and nighttime work shifts according to rules established and known by one and all. That said, schedules have become difficult to build, requiring professionals to show flexibility, often to the detriment of their personal lives [16]. When preliminarily determined and closely regulated, worktime seems out of line with occupational well-being and personal development [17]. Moreover, relationships with work appeared to have evolved and become different ; nowadays work is viewed as a means, and not a be-all and end-all [18]. In this context, new profiles for nurses have emerged, alternating work inside and outside of hospitals. In addition, economic changes such as delayed retirement have led to the coexistence in a hospital of several generations, rendering management increasingly complex insofar as marked generational specificities had to be taken into account [19, 20]. A generation is defined as bringing together individuals of similar age having experienced historic events possibly circumscribing their vision of the world [21]. As of now, generations X, Y and Z are the most widely represented, and they harbour a vision of time management and life priorities differing from those of their parents. It seems that generational differences influence expectations regarding remote working, flexibility, and the use of digital technology [22]. A generational approach consequently sheds light on the need for flexibility and autonomy of professionals belonging to the Y and Z generations [23]. In the post-pandemic era, younger generations are gravitating towards flexible, digital work arrangements and prioritising work-life balance, while older generations maintain a preference for more direct forms of communication and in-person interactions. The effective planning process necessitates the integration of hybrid models, the establishment of explicit guidelines, and the implementation of management practices that demonstrate sensitivity to the needs of different generations [24]. That said, and so as to steer clear of stereotypes on generational classifications, it is important to take into consideration elements such as religion, national history and social class [25, 20].

These different contextual elements are closely related to the question of participative management, which is aimed at associating professionals with the decision-making process, at encouraging initiatives and, more generally, at favouring self-reliance. The concept dates back to the outset of this century, when Mary Follett put forward the idea of “power with”, as opposed to “power over” [26]. Development of participative management implies and involves ingredients related to dynamic and interactive decision making and problem resolution, shared governance, empowerment, organizational transformation and dynamic communication in healthcare establishments. These different elements suggest the notion of collaborative management, which may be defined as an organizational approach through which several professionals join forces in pursuit of a common goal.

Bearing these elements in mind, how can collaborative management of nurses’ schedules positively influence the attractivity and loyalty-building in the present-day hospital sector ?

## Materials and methods

### Study design

An explanatory sequential mixed methods study was conducted [27, 28]. The quantitative method, which utilizes surveys and data, facilitates the measurement of impact (satisfaction, loyalty) and the identification of overall trends. However, this analysis does not provide a sufficient explanation for the occurrence of these effects.

The qualitative method (interviews) is instrumental in elucidating the mechanisms (sense of autonomy, recognition, work-life balance) and the organizational contexts that shape these impacts.

This study integrates the quantitative and qualitative approaches, thereby combining statistical rigor with analytical depth. This integration results in findings that are both reliable and grounded in reality.

In the initial phase, a quantitative study enabled a situational analysis in different hospital structures concerning nurses’ experience of schedule management. In the following phase, a qualitative study consisted in a series of semi-structured interviews with nurses, the objective being to measure the effects of collaborative management on the satisfaction, and investment of hospital-based nurses. The results of the quantitative study facilitated the elaboration of an interview grid for the qualitative study.

### Participants

For the quantitative study, public sector nurses working in mainland France and overseas territories were asked to participate. They were recruited from hospitals categorized as Hospital Centres or University Hospital Centres ; the latter are associated with a university, and have a greater capacity to receive patients. Nurses working in another sector (private nurses, home care nurses) were excluded from the study. All in all, 208 nurses completed the online questionnaire. Sample size was calculated by cumulative sampling [19].

For the qualitative study, 13 volunteering nurses were recruited after having given their consent and filled out the questionnaire of the quantitative study.

### Data collection

In August 2024, data were collected on nurses working in hospital centres or university hospital centres in different French regions. Questionnaires were transmitted through the communication channels of hospitals, professional networks and nursing colleges via e-mails to the relevant regional referents. Circulation of the questionnaire was ensured by “Redcap”, a secure web platform enabling data transfer on Excel.

The quantitative study is predicated on a questionnaire comprising five items. Firstly, the demographic and professional information of the participants is to be collected, including age by age group, gender, hospital setting, department, nursing experience, and region of the healthcare facility in France. Secondly, the shared timetable management system is to be implemented. The study will encompass five inquiries, including satisfaction measured on a dichotomous scale, regarding participation in timetable creation, the tools available to view the timetable, the ability to make changes to the timetable independently, and the approval of the available timetable. Thirdly, job satisfaction will be measured using a Likert scale to assess schedule management and creation. Fourthly, retention and intention to stay will be analyzed using a commitment model. The study will employ a three-question ordinal variable on a five-point scale. The final section is a space for comments on possible improvements to the management and creation of the schedule. The responses to the items will be cross-analyzed according to place of work, care sector, and age, depending on the region.The questionnaire was meticulously designed to align with the objectives of the study.

The results of the quantitative analysis, particularly the key findings, were explored in greater depth through the qualitative study. In addition, a semi-structured interview was developed for the purpose of furthering the investigation.

The 13 interviews were carried out from 27 August through 30 September 2024, either face to face or by video conference. Mean interview duration was 33 (19–45) minutes. From one stage of the research to the next, a diary was kept, the objective being to provide informative elements conducive to reflexivity and contextualization.

### Ethical considerations

On 15 July 2024, the ethics committee of the *Health Education and Promotion Laboratory* (LEPS) transmitted its official validation of the study, compliance with the ethical standards of Sorbonne Paris Nord University. Clinical trial number is not applicable.

The questionnaires were anonymized. They were accompanied by a note indicating the possibility of recontacting the researcher so as to participate in the second part of the study by interview via a dedicated e-address.

The interviews were recorded following the consent of the interviewed party and anonymized. The interview grid was revisited subsequent to study of the questionnaire responses, the objective being to render the semi-structured interviews increasingly relevant. This study adhered to the declaration of Helsinki [29].

### Data analysis

For the purposes of the quantitative study, analysis of the data retrieved from the questionnaire via Redcap was carried out using an Excel spreadsheet and “Rstudio” software for a descriptive cross-sectional analysis, which brought to bear a number of variables : age, experience, region, establishment.

#### Recoding

The “scheduling availability” variable was dichotomized as “15 days to 1 month” versus “> 1 month”. Given the heterogeneity of the “geographic zone” variable, it was secondarily recoded in binary format: Ile de France / Province. Given the specificities of the population, the “Uncertain” and “Don’t know” responses to the “loyalty-building” question » were similarly brought together.

#### Statistical analyses

Descriptive analysis verified data distribution and the existence of missing data. The small proportion of missing data led us to process them by complete case analysis. As the variable was categorical, the Fisher’s exact test was used at a *p* value of 5% during the crossed analysis.

For the qualitative study, “ATLAS.ti” software was used [18]. Analysis thématic was used. Initial coding was carried out using an inductive, followed by a deductive approach employing the following variables : job satisfaction, work-life balance, motivation, scheduling through shared or collaborative management. Axial coding was then generated, bringing together similar codes so as to form themes. By identifying recurrent words and motifs, thematic analysis led to the elaboration of thematic maps conducive to visualization of the links between the themes. Six themes emerged :

1. Schedule management : to participate or not ?
2. And if the schedule were subject to discussion ?
3. Can scheduling be conducive to work-life balance ?
4. The tools to promote schedule devising and management
5. Some reasons for nurses to remain motivated.
6. Generation X and the march of time!

Lastly, subsequent to the interviews, triangulation with a second researcher trained in research methods was develope.

#### Data integration

Analysis of the quantitative questionnaire facilitated elaboration of the interview grid for the qualitative phase. Given the quantitative results, it seemed important that the semi-structured interview specify participation in schedule management / construction. Along with the tools applied, the time management process represented a parameter to develop, while satisafaction for nursing practice called for definition.

## Results

### Participant characteristics (Table 1)

**Table 1:** Descriptive population of quantitative study

Total of nurses	208
Sexe	**women** : 86%; **men**: 14%
Generations	**Z** 1,2%. ; **Y** 28.5% ; **X** 66.5% ; **babyboomers** 3,8%
Place of practice	48% **hospital centres ;** 51% **university hospital centres ;** 1% no answer
Experience exercice	**< 1 year** :0,4%. ;**1–5 years** :17, 5% ; **6–10 years** 22,6% ; **11–15 years** : 14,5% : **>15 years** :45%

208 nurses (women: 86%; men: 14%) participated in the study; 95% of the respondents belonged to generations X and Y. In 45%, the nurses’ professional experience exceeded 15 years. As regards the nurses’ places of practice, 47% worked in hospital centres, and 51% in university hospital centres. They exercised in different, primarily medical units (44.7%), and also in operating theatres, intensive care, geriatrics, and paediatric psychiatry.

The thirteen interviews were conducted with women, of whom nine had more than 15 years of experience, one from 11 to 15 years, one from six to 10 years, and two for less than six years. Seven of them belonged to Generation X, five to Generation Y, and one to Generation Z; while nine practiced in hospital centres, the four others were employed in university hospital centres.

### Results of the quantitative study

Among the study participants, 32.7% participated in schedule devising. For 43% of those nurses, representing 13% of the study population, participation consisted in the elaboration of timetables, which they transmitted to the head nurse for validation.

Among the 32.7% who participated in schedule devising, 84% of whom worked in non-university hospital centres, 81% were satisfied. Slightly more than half of the nurses (52.4%) enjoyed some autonomy concerning choice of their work days, and the overwhelming majority (98%) were satisfied (98%).

Under French law, in accordance with established conditions, schedules may not be modified fewer than 15 days before coming into force. Schedule validation involved 60.5% of the participating nurses, 30% of whom received notice one to two months previous, and 4% six months previous; dissatisfaction among those who had their schedules validated 15 days previous was high (75%), whereas all of those (100%) who had their schedules validated three to six months previous expressed satisfaction.

The nurses who had at their disposal a yearly schedule template (67%) expressed widespread satisfaction (88%). Some (38%) had no template or other tool allowing them to visualize their schedules.

As regards the devising and management of their schedules, only 40% of the nurses were satisfied, and they described negative repercussions on their personal lives.

As regards workplace well-being, 78% of the nurses affirmed that it implied schedule devising. For 50%, participation in schedule devising was a source of satisafaction and for 57.7%, it was associated with improved job satisfaction. Among the nurses, 63.5% stated their intention to stay in place for the following two years, and 49% suggested that schedule management would strongly impact their loyalty toward their place of practice. While 41% said that schedule management would be factored into their decision to stay or not to stay, 40% declared that there was no linkage. While 44% felt that participation in the devising of their schedule affected the attractivity of their job, 69% of those who did not yet participate in the devising of their schedule expressed their readiness to do so.

No statistically relevant distinction was observed between the four age groups according to sex, type of hospital centre, hospital unit, or geographic zone. Nor was there any significant distinction according to schedule availability prior to its taking effect or the possibility of modifying the schedule in coordination with colleagues, without an intervention by the head nurse.

It seemed that the older the professionals became, they more they felt that the devising of their schedule contributed to occupational well-being.

Moreover, 65% of the persons participating in the devising of their schedules were satisfied with the devising and management, as opposed to 39% who did not participate.

Nearly half (49%) of the professionals perceived a positive influence on their work-life balance when they participated in the devising/management of their schedule, as opposed to 22% when they did not participate.

Among those who participated, for 62% the attractivity of their job was associated with devising and managing their schedule, as opposed to 36% among those who did not participate. (Table 2)

**Table 2:** Key points of the quantitative analysis

Factor	Effect on Job Satisfaction	Context/Notes
Participation in schedule elaboration and construction	Enhances job satisfaction	Active involvement in planning increases morale and engagement.
Effectiveness of schedule participation in hospital centers vs. university hospital centers	More effective in hospital centers than in university hospital centers	Organizational culture or workload differences may influence this disparity.
Schedule management	Impacts loyalty building	Effective management fosters long-term commitment and retention.
Schedule projection validated 3–6 months before implementation	Source of satisfaction	Advance planning provides stability and predictability for employees.
24 /7 access to schedule visualization	Source of satisfaction	Transparency and accessibility improve work-life balance and reduce uncertainty.
Age of nurses	More positive impact on job satisfaction as nurses grow older	Experience and evolving priorities may increase the value of flexible, well-managed schedules.

### Results of the qualitative study

“Atlas ti” software was mobilized to carry out qualitative analysis of the semi-structured interviews. All in all, 347 citations were noted ; among them, 41 units of meaning were obtained, and were sorted into 15 categories. Six themes emerged, allowing for responses to the research question : How does collaborative management of nurses’ schedules impact attractivity and loyalty building in the hospital sector ?

#### Schedule management : to participate or not?

An imposed schedule “*creates difficulties with regard to personal life and personal well-being and what with the lack of nurses*,* long sick leaves and lack of recruitment*,* we aren’t able to set it up*”. And also: “*Validations are clear enough when they’re to be programmed at six months*,* but it’s complicated when there’s no schedule*”. Participation occurs at several levels: in a schedule with the template drawn up by the head nurse and allowing for modification; in a schedule without a template established by the head nurse. Active participation in schedule devising empowers the professionals. Some teams rely on themselves for creation of schedules with templates, a form of organization allowing them to feel that they’re managing their timetables. Once annual leave proposals are validated on the quarterly template and posted, the professionals can participate autonomously in the choice of days off. Some teams build a schedule without a template with volunteer referents and subsequently submit it to the head nurse for validation: “*Each actor is responsible for filling out the empty spaces and gaps*”. In these different schemas calling for collaboration, the head nurse’s role consists in validating the team proposal.

#### And if the schedule were subject to discussion ?

Solicitations of team members by the head nurse in the framework of schedule management likewise seem to be appreciated. When the head nurse accepts feedback on the established schedule so as to avoid errors, the professionals are appreciative: “*As long as dialogue and communication continue*,* there are possibilities*”. Head nurse consilience (“convergence of evidence from different, unrelated sources”) was a repeatedly cited notion. Conversely, the impression of “being in front of a wall” exists and brings about a form of professional ill-being, particularly when the concerned parties are not informed of a change in schedule. When communication is absent, so is loyalty building, and the professionals have no qualms about leaving their current place of employment : “*Without so much as consulting me*,* they assigned me to orthopaedics … I did not like it in the least*,* and after just one month*,* I handed in my resignation*”. To top it off, apparently incoherent requests (days worked early in the year…) lending themselves to late-stage validation are perceived negatively. Drawing upon their experience, the nurses suggest that autonomously or collaboratively managed schedule devising is optimally adapted and felicitously experienced, and they deplore “*the hypocrisy of a system*” that valorises well-treatment and workplace quality of life, but does the opposite in schedule management.

#### Can scheduling be conducive to work-life balance ?

Scheduling is an indispensable projection tool : “*The more we can anticipate*,* the better it is*”, “*if (only) it could be anticipated*,* planned out*”. That much said, projection of annual leave by the institution at the outset of the year is viewed as overly distanced in time (“*As for myself*,* I find that yearly leave is determined too early*”), and as having a negative impact on personal balance.

In clinical practice, validated schedules are presented in different and divergent time frames. A schedule validated one month before taking effect remained acceptable for some (“*the timetables came out one month before*”), but at more than one month, they would be more satisfactory, especially when there were no templates. The nurses were consequently satisfied : “*We’re so glad to have two and a half months*,* it’s a pleasure to have time to organize our personal lives!”* A key element expressed by the nurses was the need for timetable flexibility and adaptability. Satisfaction predominated when it was possible, in conjunction with one’s colleagues, to modify one’s schedule : “*flexibility*,* or if one has a an appointment*,* the possibility to adjust*”. Adaptability is exemplified in schedule drawn up by the nurse herself : “*I have many colleagues in single-parent families*,* single mothers who arrange to have a maximum number of days with their children when they are not working*,* and who strive to organize themselves accordingly; a nurse can enjoy leisure when working every other weekend; she can even have a social life*,* which isn’t always the case*”. In other words, a schedule determined by the professionals themselves yields satisfaction with regard to a need for flexibility and freedom in accordance with their personal lives : “*While we put in nights on the job*,* each of us works to some extent when she wants*,* and yet we try to keep things under control*”.

Whatever the arrangement, daily management of projected schedules is considered complicated: “*It’s the same shambles everywhere and anywhere*,* and it depends a lot on the head nurse”.* Imposed schedule changes are experienced as a source of difficulties : “*I’ve already had to observe three timetables in three days*,* and believe me*,* it’s complicated*…”. For some nurses, non-projection ceases to be possible, and they end up calling it quits : “*It was a provisional schedule*,* subject to change at any time … I can no longer work under such conditions*”. A climate of tension due to schedule uncertainty is often a source of ill-being : “*a markedly negative climate in which the paramedical team is pressuring the management team by exclaiming: ‘We’re still with no schedules. I’d like to have my time off* !’”. The negative impact on workplace quality of life is altogether real.

#### The tools to promote schedule devising and management

A tool to visualize one’s schedule 24 h a day, seven days a week, seems to have become necessary, and questions were raised concerning its democratization. Some of the nurses have access to their establishment’s time management software, which enables them to visualize, generally on site, the state of their day counter. The existence of that option should reassure others, who mentioned the issue of paper passage and transcription in institutional software, which would improve schedule readability. On this subject, a specific application is used for teams enjoying autonomy in schedule creation, and it differs from the internal institutional software to such an extent that dedicated networks have been brought into being, especially by teams ensuring their own schedule management. Last but not least, the right to disconnection is possible and can be exercised by one and all, given the imperativeness of taking into account the need to “turn off and tune out”.

#### Some reasons for nurses to remain motivated

As scheduling is a source of motivation for nurses, prior to joining a team they inform themselves on timetable organization, particularly with regard to the existence of a template.

Team atmosphere and mutual support are of importance when deciding on joining and remaining in a hospital unit, as is the oft-associated impact of a given specialty : “*I knew at the time who was the head nurse with regard to the state I was in*,* and I knew the physicians who were working there*”, “*In fact I adore my colleagues*,* it’s a matter of atmosphere*”. Whatever the specialty, caregiving, technicity, pace of work and workplace autonomy are appreciated by nurses in their everyday practice. Conversely, lack of recognition, understaffing and the closing of beds have a negative impact on a nurse’s loyalty and motivation.

#### Generation X and the march of time !

During the interviews, the diverging practices of the concerned generations were mentioned by nurses of Generation X, who tended to dwell upon the differing approaches to work of their younger colleagues : “*We are now dealing with a generation that doesn’t see things in the same way; as regards scheduling*,* they cannot perform more than three days or three nights*”. Attitudes on relationship to work have been evolving, and the devising and managing of schedules are called upon to evolve accordingly : “*Working periods are really of paramount importance*,* relationship to work has changed*,* the culture is no longer the same*”. (Table 3)

**Table 3:** Key points of the qualitative analysis

Aspect	Details	Impact/Implications
Participation	Creation of schedules for nurses in hospital centres; self-directed modifications in university hospital centres. Participation allows professionals to feel free.	Enhances autonomy and job satisfaction.
Schedule Management	Fosters well-being (work-life balance) or ill-being (leading to nurse departure). Flexible and adaptable scheduling is of major importance for nurses.	Critical for retention and morale.
Schedule Projection	Planning annual leave one year in advance is difficult; validation two to six months prior is satisfying.	Advance validation improves satisfaction and reduces stress.
Schedule Visualization	24/7 access to schedule visualization is a source of satisfaction; calls for further development while respecting the right to disconnection.	Increases transparency and work-life balance.
Social Networks	Used as tools in schedule devising and management by current teams.	Facilitates communication and collaboration.
Motivation and Atmosphere	Scheduling motivates nurses, but team and specialty-related atmosphere are equally important and indispensable.	Holistic approach to job satisfaction is essential.
Generational Differences	Generation X perceives a relationship to work differently from younger generations.	Highlights the need for tailored management strategies across generations.

### Summarized analysis of the quantitative and qualitative studies

The study permitted assessment of satisfaction, commitment related to collaborative management of schedule devising and management among nurses in France practicing in the public hospital sector. Motivation is a theme that emerged from the qualitative study. Collaborative management and empowerment in timetable organization and modification are factors of attractivity and loyalty building in this population.

**Fig. 1:**
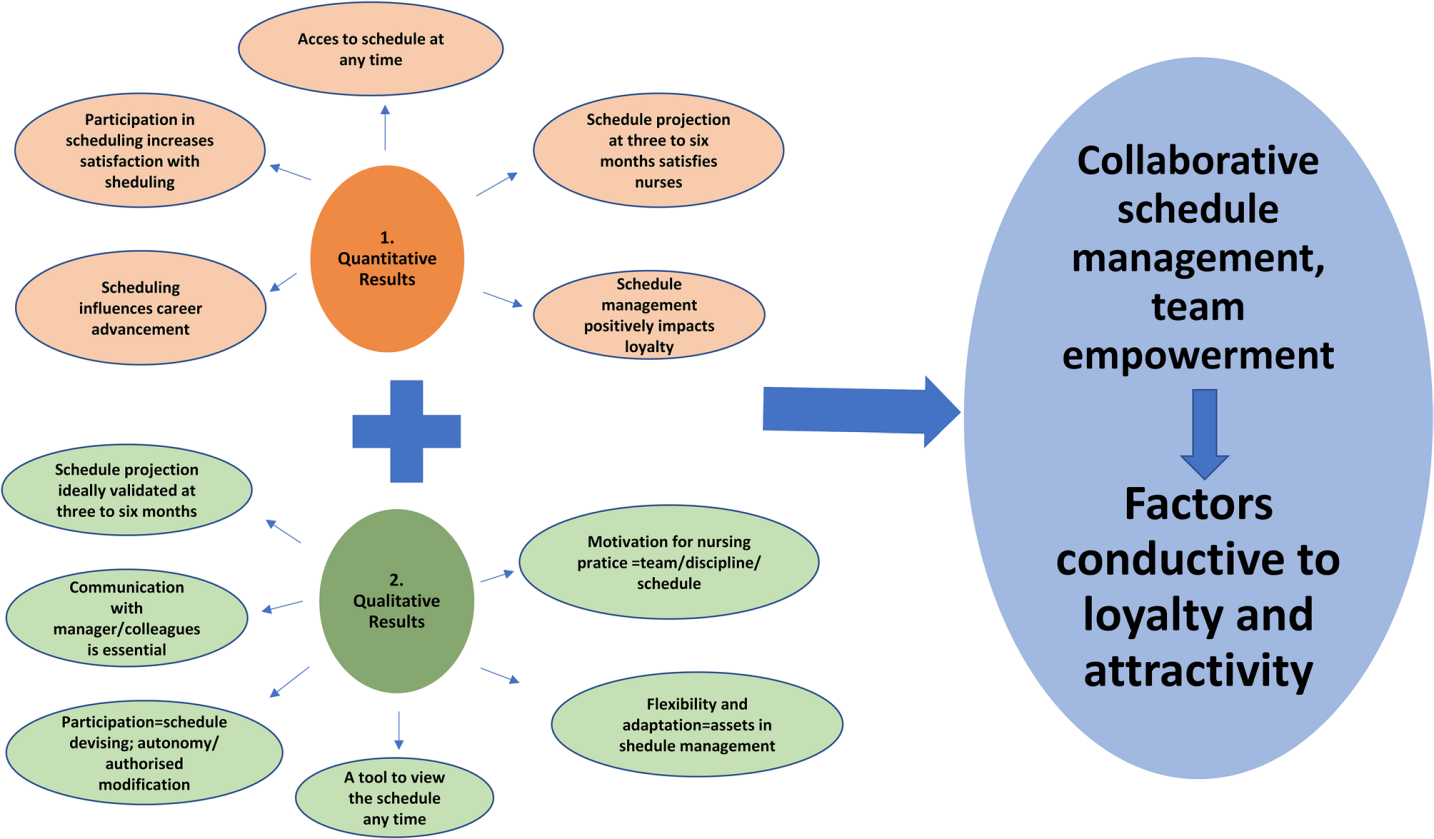
General summary of the study

This review of the different forms of schedule devising and management described by nurses throughout the study show that there exists no uniform practice in French hospital centres in the devising and management of nurses’ schedules. (Fig. 1)

## Discussion

This study on a hospital-based nursing population has raised the question of the effects of collaborative scheduling management on attractivity and loyalty building. By applying a mixed explanatory approach, collaborative scheduling management and team empowerment were found to appear as factors favouring attractivity and loyalty building.

### Participation in scheduling

The quantitative and qualitative results combine to show the job satisfaction is enhanced when nurses actively participate in schedule devising and management. More generally, they reinforce the concept of collaborative management, an approach that optimizes performance of teams by empowering them, by endowing them with decision-making power, and by favouring investment and creativity. Collaborative management is based on shared decisional authority, transparency and collaborator involvement in the decision-making process [30]. With this in mind, the drawing up of nurses’ schedules by their own teams, which is currently a widespread practice among the concerned parties, is signally conducive to shared decision-making. It counteracts the sense of powerlessness and injunction experienced in some of the contexts described in this study. Collaborative management is a participation-based conception through whose implementation each and every professional in a team is an active member duly recognized for his or her skills and knowledge. More broadly, it is at once a process stemming from a shared vision, and a form of participative activity in which all of the actors are considered as experts, and in which their participation is based on what they know rather than the roles they play or the interests they represent [31]. While this concept has been evolving since the 1970s, its implementation with regard to schedule devising and management does not seem to have been satisfactorily successful, particularly in large-scale institutions such as university hospital centres. The pyramidal hierarchies that still predominate in these establishments are likely to constitute an obstacle. In this context, collaborative management, which is characterized by a director’s determination to activate and benefit from collective intelligence, does not seem to have taken root [30]. The concept of self-managed teams, as opposed to collaborative management and collaborative planning, refers to groups that are responsible for a “task as a whole,” with genuine autonomy over the division of labor, methods, and, in some cases, planning [32]. As previously delineated, the efficacy of these entities is contingent upon the nature of the task, the prevailing organizational context, the hierarchical structure, and the individual competencies of its constituents. According to Magpili and Pazos, self-managed teams are teams endowed with genuine collective autonomy to organize and carry out interdependent work [33]. This also sheds light on teams that embody this self-management, a path toward empowerment.

### Team empowerment

Nurses’ motivation is generated by belonging to a team and by overall team atmosphere. A shown by Siebert et al. in 2011, collective motivation has a positive impact on personal motivation, and vice versa [34].

Work-life balance and schedule projection are essential elements emerging from the qualitative and quantitative results of the present research. Given the satisfaction arising from participation in scheduling, implementation of the empowerment it involves can be envisaged, as fulfilment of a wish voiced by professionals of all generations, who are determined to be recognized with their acquired skills and to be provided with autonomy in their field of action. So it is that empowerment can be considered as a fundamental need, as part and parcel of a continual relational process during which the organization adjusts its degree of regulation and control to the extent that a worker mobilizes his or her subjectivity (and vice versa) [35]. In other words, empowerment consists in providing resources, support and freedom to make decisions and manage one’s own activity. When put into practice, it builds trust between collaborators and the self-empowered employee, redounding positively on individual as well as collective expertise and accountability.

However, empowerment is far from obvious; it is a process requiring forethought and projection. It results from ongoing interactions and negotiations that lead to determination of the precise scope between instrumental contingencies and desire for emancipation, between autonomy required and autonomy conquered [35]. These distinctions could help to explain the differences between centres with regard to participation in schedule management, and may raise questions concerning the respective roles of skills and autonomy. All told, empowerment is a form of acculturation necessitating apprenticeship ; if it is not attempted, backsliding is likely to ensue.

### A capacitating environment

Flexible and adaptable scheduling is an exigency permeating this study and a parameter for professional satisfaction, investment and loyalty. A head nurse viewed as conciliatory can be the manager recognized as apt to bring about the expected flexibility and adaptability. Capacitating environment is a key concept, connecting empowerment and collaborative management. In some cases, the front-line manager assumes a key role in promoting awareness of the environment and implementing empowerment on the scale of a given unit. Capacitating environments are spaces in which individuals have the option of tapping into and drawing upon resources allowing them to learn and evolve [36]. They are spaces in which the capacity to act exists. The more an environment is conducive to developing one’s capacities, the more it is considered as capacitating [37]. A propitious setting enables professionals to individually and collectively activate their capacities, thereby broadening their horizons and creating new know-how and knowledge [38]. A capacitating organization allows individuals to learn, to construct their apprenticeship and manage their activities. It is conducive to practice-centred discussion and debate, through which the organization continually evolves. Capacitating spaces are sites for implementation of collaborative management and empowerment, which are factors for attractivity and loyalty building among nurses in a hospital setting. Transformational leadership steering professionals toward an “elsewhere” in which they are personally recognized serves as a spur for meaningful change. Since 1980, various models of collaborative management have appeared: the “Magnet Hospital” in the United States, the ARIQ model in Belgium (Attraction, Retention, Involvement of nurses and Quality of care), and “Cluster QVT” (quality of work life) ; these approaches enable healthcare establishments to effectively integrate new modalities of collaborative management [39]. Once embedded in institutional or departmental policy, these initiatives support the front-line manager in her missions within organizations, one of them being time management (9). The relevant concepts bring together collaborative management and empowerment [40], which have been recognized and highlighted in our study as factors favouring loyalty and attractivity in the devising and management of nurses’ schedules.

### Limitations of the study

While cumulative sampling was applied in a given time frame, representation of the different hospital centres in each region was not complete. Paralleling of the relevant institutional policies would probably have yielded some responses on differences between the centres. In order to guarantee a thorough and succinct approach, it has been decided that the questionnaire will be adapted to the study. The social representations associated with the researcher constitute a study bias ; that said, triangulation was carried out by incorporating the viewpoints of a confirmed researcher and a co-researcher.

In the final analysis, this study demonstrates that in the devising and management of nurses’ schedules, empowerment and collaborative management in the hospital sector are factors conducive to loyalty and attractivity. Indeed, empowerment favours job satisfaction and, in a win-win situation, reinforces motivation and investment. On the other hand, our research on time management did not shed light on the generational factor.

### Futures directions

Given the study’s findings, it is essential to position schedule management as a central component in the strategies aimed at attracting and retaining nurses within healthcare institutions. To further develop this focus, additional research should be conducted among local and senior managers in French hospitals and university hospitals, particularly examining human policies and care plans in consultation with Directors of Nursing and Human ressource Directors. The study underscores the critical role of frontline management, especially in the organization and scheduling of nurses’ timetables, which are vital tools for achieving **a** work-life balance. Ultimately, healthcare managers emerge as key pillars whose recognition and support are indispensable for successfully retaining and attracting nursing talent in the hospital sector.

### Theoretical and pratical contributions of the study

This study explores a subject that has received minimal attention : the management of work schedules in the healthcare sector. Nevertheless, it remains a pivotal concern for healthcare administrators and the structuring of healthcare systems. This phenomenon enables us to: it is imperative to develop a more profound comprehension of the methodologies employed in the administration of work schedules and their ramifications on both staff members and patients.It is imperative to demonstrate that this frequently disregarded subject is pivotal to enhancing quotidian working practices.

The study puts forth concrete solutions to enhance shift planning in France, with a focus on two primary domains. The training of healthcare managers is of paramount importance. The development of their collaborative leadership skills is essential for the purpose of involving teams more closely in the creation of shifts plans. The adoption of a participatory management approach, which involves listening to the needs of healthcare staff and adjusting shift plans accordingly, is also crucial. The utilization of suitable digital instruments is imperative for the effective management of shift operations. The implementation of collaborative software facilitates the coordination of personnel, ensuring the timely communication of their availability. Moreover, the automation of specific tasks contributes to the optimization of temporal efficiency and the reduction of errors. The objective of these solutions is to enhance the working conditions for healthcare personnel and optimize the organization of care.

## Conclusion

This study demonstrates that among hospital nurses, time management by collaborative management and empowerment constitutes a parameter of attractivity and loyalty warranting further development. The existing multitude of schedule devising and management schemes in hospitals and hospital units are far from being uniform and fully satisfactory. As of now, however, it is practicable to envision inclusion of a time management axis that would contribute to attractivity and the building of professional loyalty.

To deepen our exploration and continue to advance, it would be interesting to undertake additional research involving front-line and senior managers in French hospitals, the objective being to obtain precise information on the policy orientations of different management teams.

In this study, the effects of front-line management and its role as a fulcrum have been identified as crucial factors of attractivity and loyalty ; this is particularly the case regarding the devising and management of nurses’ schedules, which may serve as tools helping to anchor personal and professional life. With these considerations in mind, the head nurse remains a pillar to be recognized and supported in the process of enhancing attractivity and building loyalty among nurses in the hospital sector.

## Electronic Supplementary Material

Below is the link to the electronic supplementary material.

Supplementary Material 1

Supplementary Material 2

## Data Availability

The data that support the finding of this study are available upon reasonable request from the authors and with permission from ethical bodies.This study adhered to the déclaration of Helsinki.

## Abbreviations

ARIQ: Attraction, Retention, Involvement of nurses and Quality care
LEPS: Health education and Promotion Laboratory
QVT: Quality of work life
WHO: World Health Organization
