# Supplementary material for: The effect of schedule management empowerment on hospital attractivity and nurses’ loyalty: a mixed method study
Source: BMC Nurs. 2026 May 25;25:646. doi: 10.1186/s12912-026-04713-w (PMC13386555; doi:10.1186/s12912-026-04713-w)
Supplement: Supplementary file 1 — Supplementary Material 1 [file 12912_2026_4713_MOESM1_ESM.pdf]

Semi-structured interview guide for studying the management of nurses' schedules in the hospital sector and their retention

Presentation of the study

I invite you to participate in a study on the management of nurses' schedules in the hospital sector and their retention. This study is under my responsibility, Sophie PAJOUX. Its objective is to visualize the effects of the management and construction of schedules in the hospital sector today on the retention of nurses in light of the current context.
Your participation is entirely voluntary. An email address is available for any comments or feedback at any time: gaia.rechercheM2RIS@gmail.com This study consists of two distinct parts. The second part consists of semi-structured interviews, which is what we are conducting today. Each interview will take approximately 30 minutes. The interview will be recorded for subsequent analysis. All data will be completely anonymized.

1. Demographic information

Can you briefly tell me about your professional background?

2. Schedule management/schedule description/participation/satisfaction

Can you describe how your schedule is currently organized?

Can you explain how it is structured?

Can you tell me if you are satisfied with the current system for creating and managing your schedule?

Keywords: approved schedule - defined time for approved schedule - framework - ideal time for satisfaction - available tools - projection - wishes

Was it the same in your previous jobs? Better or worse?

Conclusion

Finally, what factors enable you to remain in a unit?

What attracts you to a particular position? To a particular unit?

Do you have any questions or comments following this interview?

Thank you very much for your participation.
