# Supplementary material for: The effect of schedule management empowerment on hospital attractivity and nurses’ loyalty: a mixed method study
Source: BMC Nurs. 2026 May 25;25:646. doi: 10.1186/s12912-026-04713-w (PMC13386555; doi:10.1186/s12912-026-04713-w)
Supplement: Supplementary file 2 — Supplementary Material 2 [file 12912_2026_4713_MOESM2_ESM.pdf]

Summary table of the themes, categories, and codes that emerged from the thematic analysis of the 13 interviews conducted as part of the qualitative study entitled « The effect of schedule management empowerment on hospital attractivity and nurses' loyalty: a mixed method study »

THEMES	CATEGORIES	CODES	NUMBER OF CITATIONS	NUMBER OF NURSE
Private life / professional life: planning	scheduling	• IDE highlights the ever-changing schedules	6	5
		• IDE emphasizes the professionalism required for planning	1	1
		• IDE believes that the key to improvement lies in increasing staffing levels in the units	4	3
	flexibility	• ide notes that the schedule can interfere with personal life	5	3
		• ide explains that the schedule is the same mess everywhere	3	2
		• ide expresses the need for flexibility in the schedule	9	6
		• ide highlights the constraints associated with the profession and the hospital structure	1	1
	projection	• The user expresses satisfaction with the 2-month	9	5
		• The user mentions a 6-month schedule for cases	1	1
		• The user mentions the requested 1-year schedule	13	7
		• The user mentions the schedule forecasting tool	21	13
total	3	11	**73**	
Creating the schedule: to participate or not?	autonomy and collaboration	• ide explains the collaborative nature of the scheduling	39	11
		• ide expresses great satisfaction with the self-managed schedule	22	11
		• ide emphasizes the importance of taking an active role	12	7
	mandatory schedule	• ide expresses dissatisfaction with the imposed schedule	19	10
	respects wishes	• The respondent notes the impact of the schedule on their overall satisfaction	19	10
		• The respondent identifies the schedule as a source of anxiety and distress	5	4
total	3	6	**116**	
If we were to discuss the schedule	comunication	• ide mentions respect stemming from familiarity with the staff	6	5
		• ide explains that their resignation was related to how the schedule was managed	9	4
		• ide points out the hypocrisy of the system	8	4
		• ide notes that their requests were not being heard	26	9
	context of the schedule	• ide highlights the manager's role in setting schedule rules and deadlines	16	9
		• ide outlines the conditions for managing the schedule	15	6
**total**	2	6	**80**	
Tools for managing/creating the schedule	existing tools	• ide mentions the use of an internal team tool	7	7
		• ide mentions self-management via software	5	3
		• ide mentions a network-based system for schedule management	5	4
	24/7 view	• ide highlights the benefits of the 24/7 computerized schedule	12	8
		• ide says that the work email is sufficient for tools	2	1
		• ide says she is not in favor of having the schedule at home	3	2
**total**	2	6	**34**	
Motivation for nursing practice	various reasons	• ide cites the schedule as a source of motivation	6	5
		• ide cites intellectual stimulation, recognition, and	2	1
		• ide cites teamwork and the specialty as sources of motivation	16	9
		• ide states that they work based on their compensation	2	2
		• ide explains reasons for leaving the hospital sector	5	4
		• ide mentions that the appeal of a new position is the new experience	1	1
**total**	1	6	**32**	
Generation X and changes over time	professional insights	• ide says they have adapted to it because of their age	1	1
	in relation to the younger generation	• ide expresses a difference in collaborative behavior among the younger generations	3	1
		• ide expresses a difference in working with the younger generation	1	1
		• ide expresses a difference in working with the younger generation	2	2
	societal changes	• ide notes that social norms have changed, and attitudes toward work have changed	1	1
	place de l institution	• ide notes that the institution offers incentives to attract young people, but what about the older staff?	1	1
**total**	**4**	**6**	**9**	
